# Remote ischemic conditioning after stroke: Research progress in clinical study

**DOI:** 10.1111/cns.14507

**Published:** 2023-11-06

**Authors:** Bin Jiang, Xiaojie Wang, Jianping Ma, Aminah Fayyaz, Li Wang, Pei Qin, Yuchuan Ding, Xunming Ji, Sijie Li

**Affiliations:** ^1^ Department of Neurology Shenzhen Qianhai Shekou Free Trade Zone Hospital Shenzhen China; ^2^ Department of Neurosurgery Wayne State University School of Medicine Detroit Michigan USA; ^3^ Department of Neurology, Xuanwu Hospital Capital Medical University Beijing China; ^4^ Beijing Institute of Brain Disorders, Collaborative Innovation Center for Brain Disorders Capital Medical University Beijing China; ^5^ Department of Emergency, Xuanwu Hospital Capital Medical University Beijing China; ^6^ Beijing Key Laboratory of Hypoxic Conditioning Translational Medicine, Xuanwu Hospital Capital Medical University Beijing China

**Keywords:** intracranial atherosclerotic stenosis, ischemia/reperfusion injury, neuroprotection, remote ischemic conditioning, stroke, vascular cognitive impairment

## Abstract

**Background and Purpose:**

Stroke is a leading cause of global morbidity and mortality, indicating the necessity and urgency of effective prevention and treatment. Remote ischemic conditioning (RIC) is a convenient, simple, non‐intrusive, and effective method that can be easily added to the treatment regime of stroke patients. Animal experiments and clinical trials have proved the neuroprotective effects of RIC on brain injury including (examples of neuroprotective effects). This neuroprotection is achieved by raising brain tolerance to ischemia, increasing local cerebral blood perfusion, promoting collateral circulations, neural regeneration, and reducing the incidence of hematomas in brain tissue. This current paper will summarize the studies within the last 2 years for the comprehensive understanding of the use of RIC in the treatment of stroke.

**Methods:**

This paper summarizes the clinical research progress of RIC on stroke (ischemic stroke and hemorrhagic stroke (HS)). This paper is a systematic review of research published on registered clinical trials using RIC in stroke from inception through November 2022. Four major databases (PUBMED, WEB OF SCIENCE, EMBASE, and ClinicalTrials.gov) were searched.

**Results:**

Forty‐eight studies were identified meeting our criteria. Of these studies, 14 were in patients with acute ischemic stroke with onset times ranging from 6 h to 14 days, seven were in patients with intravenous thrombolysis or endovascular thrombectomy, 10 were in patients with intracranial atherosclerotic stenosis, six on patients with vascular cognitive impairment, three on patients with moyamoya disease, and eight on patients with HS. Of the 48 studies, 42 were completed and six are ongoing.

**Conclusions:**

RIC is safe, feasible, and effective in the treatment of stroke. Large‐scale research is still required to explore the optimal treatment options and mechanisms of RIC in the future to develop a breakthrough in stroke prevention and treatment.

## Graphical abstract

Flow diagram for literature search and screening process.

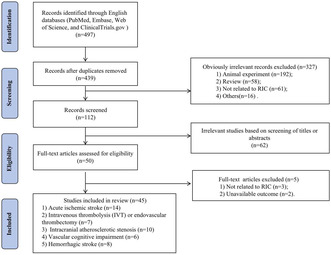

## 1 INTRODUCTION

Stroke is the leading reason for morbidity and mortality globally, with high recurrence rates. Ischemic strokes account for 80% of cases, while hemorrhagic stroke (HS) accounts for 10%–15% of cases in European and American countries and more than 20% of cases in China.^1^ Stroke has become the third leading cause of disability and the second leading cause of death worldwide.^2^ In addition, the incidence and mortality of stroke increase with age; therefore, it is important to strengthen the prevention and therapeutic strategies.^3^ Currently, the safe and useful treatments for acute ischemic stroke (AIS) are reperfusion strategy with intravenous thrombolysis (IVT) and endovascular therapy (EVT).^4^, ^5^ However, there are still a significant portion of patients who cannot achieve good clinical outcomes because of the patient's eligibility for thrombolysis, presence of contraindications, the short therapeutic time window after ischemic stroke, and limited medical resources. A study published in the journal *Lancet* in 2022 showed that the overall IVT rate for AIS in China during 2019–2020 was 5.64%.^6^ In addition, serious complications such as intracranial hemorrhage, cerebral edema, and ischemia–reperfusion (I/R) injury remain after IVT and EVT.^5^, ^7^ Hemorrhagic stroke includes intracerebral hemorrhage (ICH) and aneurysmal subarachnoid hemorrhage (aSAH). Specific breakthrough therapies for HS have been elusive, and the current treatment options include reduction in intracranial pressure, maintenance of blood volume, removal of hematoma, and coagulopathy reversal. However, only a small portion of patients have benefitted from it, with HS being designated as the most difficult type of stroke to treat.^8^ Therefore, there is an urgent need for better protective measures to effectively reduce the incidence and improve the prognosis of stroke patients.

Remote ischemic conditioning (RIC) can be easily applied to stroke patients due to its convenience, simplicity, noninvasiveness, and effectiveness.^9^, ^10^, ^11^, ^12^ Cumulative evidence has shown the brain‐protective effects of RIC are due to humoral, neural, and immune pathways that enhance the brain tolerance to I/R injury, increase cerebral blood flow, promote nerve regeneration, and the establishment of collateral circulation vessels, clear hematomas, and reduce cerebral ischemia and hypoxic injury.^13^, ^14^, ^15^, ^16^, ^17^, ^18^, ^19^ Currently, RIC is commonly considered as an innovative strategy in brain protection. The cerebral protective effects of RIC have been systematically studied, but mostly in relation to ischemic stroke; no review has been generalized to stroke, and previously published reviews have not included the most recent studies in the last 1 to 2 years. Therefore, we review the latest clinical research of RIC in the management of stroke.

## 2 METHODS

Through November 2022, we conducted searches on databases (PUBMED, WEB OF SCIENCE, EMBASE, and ClinicalTrials.gov) limited to English reports with the search terms: “ischemic conditioning,” “remote ischemic conditioning,” “ischemic preconditioning,” “ischemic perconditioning,” “ischemic postconditioning,” “stroke,” “ischemic stroke,” “cerebral infarction,” “hemorrhagic stroke,” “hematencephalon,” “cerebral hemorrhage,” and “subarachnoid hemorrhage.” The final sources chosen were selected based on their correlation to the range of this review.

## 3 CONCEPTS AND TYPES OF RIC

Remote ischemic conditioning includes remote ischemic preconditioning (RIPreC), remote ischemic perconditioning (RIPerC), and remote ischemic postconditioning (RIPostC). RIPreC means that a remote organ or tissue is given repeated I/R training before ischemia or reperfusion occurs in the target organ.^20^ RIPerC is defined as a remote organ or tissue that is given repeated I/R training during ischemia or reperfusion to protect it from ischemia.^21^ RIPostC is when a remote tissue or organ is given repeated I/R training to protect it after a prolonged period of ischemia and reperfusion.^22^ In clinical practice, it is sometimes difficult to distinguish between specific types of RIC due to the difficulty of describing the time of application, which is usually described uniformly by RIC, as well as various combinations of types. After years of development, the specific techniques of RIC are mainly to use a sphygmomanometer cuff that is tied to the upper or lower limb, then rapidly inflated and pressurized in order to block the arterial blood supply of the limb, which mimics a transient ischemic state, and then subsequently to release the pressure in the cuff bag to restore blood perfusion. A cycle is usually 3–5 min of compression followed by 5 min of relaxation, with pressures generally above 200 mmHg or greater than 30 mmHg of systolic pressure. Each training session consists of three to five cycles, one to two times per day, with the longest duration of training lasting up to 108 months (about 9 years). Repeated, transient ischemic stimulation of organs or tissues by RIC can stimulate endogenous protective mechanisms such as the neural, humoral, and immune pathways of the body, resulting in the adaptation to ischemia in vital organs or tissues other than that organ or tissue, and thus improving their ability to tolerate ischemic injury.^23^

## 4 CLINICAL APPLICATION OF RIC IN STROKE

Table 1 shows the status of clinical research on RIC in the treatment and prevention of stroke. Forty‐eight studies were identified. Of these, 14 studies were in patients with AIS with onset times ranging from 6 h to 14 days. Seven studies were in patients with IVT or EVT, 10 studies were in patients who had intracranial atherosclerotic stenosis (ICAS), six studies for participants with vascular cognitive impairment (VCI), three studies were on patients with moyamoya disease, and eight studies were patients with HS. Of these 48 studies, 39 were completed and six were ongoing. In the following paper, we present a systematic review of the safety, feasibility, and efficacy of RIC compared with conventional stroke treatments in patients

**TABLE 1:** Clinical studies of remote ischemic conditioning in the prevention and treatment of stroke.

Study	Stroke model	Type	Sample size (*n*, RIC/control)	RIC organ	RIC pressure (mmHg)	Control pressure (mmHg)	Times/day	Cycle	Time (days)	Primary outcomes	Main results	Status
Patients with acute ischemic stroke (AIS)												
Wang et al. (2022)^31^	AIS within 48 h of onset	RIPostC	42/44	Bilateral upper arm	180	60	1	5 × 5 min × 3 min 40 min	7	8 days: NIHSS, mRS, absolute platelet, platelet aggregation	Positive	Complete
Landman et al. (2023)^29^	AIS in the past 24 h	RIPostC	40/48	Non‐paretic upper arm	200	<50	2	4 × 5 min × 5 min 40 min	4	4 days: mRS, NIHSS	Negative	Complete
Ishizuka et al. (2022)^36^	AIS within 48 h of onset	RIPostC	200/200	The thigh of the unaffected side	200 or 180	50	1	4 × 5 min × 5 min 40 min	3–7	90 days: mRS, NIHSS, MACE	No data	Ongoing
Chen et al. (2022)^30^	AIS within 48 h of onset	RIPostC	922/971	Bilateral upper limbs	200	–	2	5 × 5 min × 5 min 50 min	10–14	12 days: NIHSS 90 days: mRS, MACE, incidence of neurological deterioration, proportion of death	Positive	Complete
Han et al. (2021)^35^	AIS within 24 h of symptom onset or exacerbation	RIPostC	20/20	Both arms	200	60	1	5 × 5 min × 5 min 50 min	14	12 days: Infarct volume 90 days: mRS, NIHSS, BI, walking ability, safety, adverse event	No data	Ongoing
Diamanti et al. (2021)^94^	AIS within 9 h of onset	RIPostC	40/40	Non‐paretic arm	>SBP 20	0	1	4 × 5 min × 5 min 40 min	1	24 h/72 h: NIHSS, HIF‐1α, HSP27	No data	Ongoing
Wei et al. (2020)^33^	AIS within 14 days or less	RIPostC	57/49	Healthy upper arm	200	Diastolic blood pressure	1	4 × 5 min × 5 min 40 min	30	7 days/90 days: mRS, NIHSS, BI, HRV	No data	Complete
Purroy et al. (2020)^34^	Patients with suspected acute stroke identified in the ambulance	RIPerC	286/286	Unilateral upper arm	200	0	1	5 × 5 min × 5 min 50 min	1	1 day: NIHSS, serious adverse events, sICH, acute infarct volume 5 days/90 days: acute infarct volume, mRS, NIHSS, sICH, serious adverse events, EQ‐5D	No data	Ongoing
Pico et al. (2020)^26^	AIS within 6 h of onset	RIPerC	93/95	The thigh of the unaffected side	>SBP 110	0	1	4 × 5 min × 5 min 40 min	1	24 h: mRS, NIHSS, BI, Percentage change in brain infarction volume 90 days: mRS, NIHSS, BI, MACE	Negative	Complete
Landman et al. (2023)^29^	AIS in the past 24 h	RIPostC	40/48	Non‐paretic upper arm	200	50	2	4 × 5 min × 5 min 40 min	4	4 days: infarct size 90 days: mRS, NIHSS	Negative	Complete
England et al. (2019)^25^	AIS within 6 h of onset	RIPostC	31/29	Unilateral upper arm	>SBP 20	30	3	4 × 5 min × 5 min 40 min	3	90 days: mRS, NIHSS, BI, MACE, SDS, EQ‐5D, TICS‐M, CRP, S100ß, MMP‐9, NSE, HSP‐27, mood (Zung), cognition (TICS‐M), quality of life (EuroQoL)	Positive	Complete
Li et al. (2018)^93^	AIS in the past 24–72 h	RIPostC	29/31	Unilateral upper arm	>SBP 20	30	1	4 × 5 min × 5 min 40 min	14	90 days: mRS, NIHSS, cerebral infarction volume, rCBF, rMTT	Positive	Complete
England et al. (2017)^27^	AIS in the past 24 h	RIPostC	13/13	Unilateral upper arm	>SBP 20	30	1	4 × 5 min × 5 min 40 min	1	mRS, NIHSS, BI, MACE, SAP, SDS, EQ‐5D, MMSE, CRP, S100‐ß, MMP‐9, TnT, HSP‐27	Positive	Complete
Liu et al. (2018)^95^	Acute minor ischemic stroke (AMIS)/TIA within 14 days	RIPreC	165	Bilateral upper arm	200	–	2	5 × 5 min × 5 min 45 min	90	30 days: mRS, NIHSS, BI, MACE 90 days: mRS, NIHSS, Barthel Index, MACE, recurrent ischemic stroke	No data	Ongoing
Patients with intravenous thrombolysis (IVT) or endovascular thrombectomy (EVT)												
Guo et al. (2023)^47^	Patients with AIS who underwent EVT	RIPostC	249/249	Upper arm of the unaffected	200	60	2	4 × 5 min × 5 min 40 min	7	24 h/7 days/30 days: mRS 90 days: mRS, NIHSS, BI	No data	Ongoing
Abuduxukuer et al. (2023)^96^	Patients with AIS who underwent IVT	RIPostC	279/279	The arm of the unaffected side	200	60	2	4 × 5 min × 5 min 40 min	14	90 days: mRS, mortality, adverse events	No data	Ongoing
He et al. (2020)^45^	Patients with AIS who underwent IVT	RIPostC	25/25	Healthy upper limb	200	60	2	4 × 5 min × 5 min 40 min	1	24 h: BP, hs‐CRP 90 days: mRS, NIHSS, adverse events, HT, sICH	Positive	Complete
An et al. (2020)^46^	Patients with AIS who underwent IVT	RIPostC	34/32	Both arms	180	0	2	5 × 3 min × 5 min 40 min	8–14	90 days: mRS, NIHSS, BI, S100‐β, VEGF, bFGF, MMP9, OH1, BDNF, HSP, sICH	Positive	Complete
Che et al. (2019)^43^	Patients with AIS who underwent IVT	RIPostC	15/15	Both arms/healthy upper limb	200	–	2	5 × 5 min × 5 min 50 min	6	7 days: HT 30 days/90 days: mRS, NIHSS, BI, stroke recurrence	Positive	Complete
Zhao et al. (2018)^44^	Patients with AIS who underwent EVT	RIC	20	Unilateral upper arm	200	–	1	4 × 5 min × 5 min 40 min	7	mRS, NIHSS, BI, sICH, MACE	Positive	Complete
Hougaard et al. (2014)^13^	Patients with AIS who underwent IVT	RIPerC	247/196	Unilateral upper arm	>SBP 25	0	1	4 × 5 min × 5 min 40 min	1	24 h/30 days/90 days: volume of penumbral salvage, NIHSS, mRS	Neutral	Complete
Patients with Intracranial atherosclerotic stenosis (ICAS)												
RICA (NCT02534545)^56^	AIS within 30 days/TIA within 15 days	RIC	1517/1516	Bilateral upper limb	200	60	1	5 × 5 min × 5 min 50 min	365	Time to first occurrence of AIS, 365 days: mRS, NIHSS, BI, adverse events	Negative	Complete
Li et al. (2022)^54^	Patients with the symptomatic internal carotid artery (ICA) or middle cerebral artery (MCA)	RIC	131	Bilateral upper limb	200	–	1–2	5 × 5 min × 5 min 50 min	The compliance of RIC was 95.6 ± 3.7%	8.8 years (range, 3–14 years): recurrent ischemic cerebrovascular events, MACE, mortality	Positive	Complete
Liu et al. (2021)^97^	AMIS/TIA within 14 days	RIPreC	162	Bilateral upper arm	200	–	2	5 × 5 min × 5 min 45 min	90	30 days: mRS, NIHSS, BI, MACE 90 days: mRS, NIHSS, BI, MACE, recurrent ischemic stroke	Positive	Complete
Zhao et al. (2017)^55^	Patients with severe carotid artery stenosis (CAS) prior to carotid artery stenting	RIPreC	63/63/63	Bilateral upper arm	200	60	2	5 × 5 min × 5 min 50 min	14	14 days: NSE and S‐100B, hs‐CRP, new ischemic DWI lesions, 6 months clinical events	Positive	Complete
Wei et al. (2016)^53^	Patients with sICAS	RIPostC	50/50	Bilateral upper arm	180	0	2	5 × 3 min × 5 min 40 min	180	180 days: the mean change in collateral circulation, VEGF, bFGF, NIHSS, ADL	Positive	Complete
Li et al. (2015)^52^	Patients with unilateral MCAS	RIPreC	10/24	Nondominant arm	200	200	1	5 × 5 min × 5 min 50 min	1	TOI, BP, HR, MFV, pain	Positive	Complete
Meng et al. (2015)^15^	Octo‐and nonagenarians in sICAS	RIPostC	30/28	Bilateral upper arm	200	30	2	5 × 5 min × 5 min 50 min	180	BP, HR, local skin status 30 days/180 days: NIHSS, mRS, MACE, hs‐CRP, leukocyte count, fibrinogen, D‐dimer, PAI‐1, TPA, new stroke lesion on MRI/DWI	Positive	Complete
Healy et al. (2015)^57^	Patients with carotid endarterectomy (CEA)	RIPreC	21/24	Healthy upper limb	200	0	1	4 × 5 min × 5 min 40 min	1	Clinical endpoint: MACE, postoperative complications.	Positive	Complete
Meng et al. (2012)^14^	Patients with sICAS	RIPreC	38/30	Bilateral upper arm	200	0	2	5 × 5 min × 5 min 50 min	300	30 days/90 days/300 days: NIHSS, mRS, PSV, stroke recurrence rate, cerebral perfusion status	Positive	Complete
Walsh et al. (2010)^58^	Patients with CEA	RIPreC	34/36	Legs	Pedis artery completely obliterated	0	1	1 × 10 min	1	Postoperative deterioration in saccadic latency, troponin I, adverse cardiac event	Neutral	Complete
Patients with moyamoya disease (MMD)												
Li et al. (2017)^65^	Patients with pediatric MMD	RIC	30/30	Bilateral upper arm	>SBP 50	30	2	5 × 5 min × 5 min 50 min	7	3 months: cerebral perfusion status	No data	Ongoing
Ding et al. (2019)^63^	Patients with MMD	RIC	30	Bilateral upper arm	200	–	2	5 × 5 min × 5 min 50 min	Long‐term	Safety, incidence of recurrent ischemic events, PGIC scales	Positive	Complete
Xu et al. (2022)^64^	Patients with MMD	RIC	18/16	Bilateral upper arm	200	0	2	5 × 5 min × 5 min 50 min	365	1‐year: the mean cerebral blood flow (mCBF) improvement ratio	Positive	Complete
Patients with vascular cognitive impairment (VCI)												
Poalelungi et al. (2021)^98^	AIS/signs suggestive of AIS within 24h	RIPostC	18/22	The arm of the unaffected side	180	30	2	5 × 3 min × 5 min 40 min	5	3~4 days: infarct volume 180 days: infarct volume, NIHSS, mRS, ADL, MoCA, PHQ‐9	Positive	Complete
Li et al. (2020)^32^	Patients with poststroke cognitive impairment	RIPostC	24/24	Unilateral upper arm	>SBP 20–30	30	1	4 × 5 min × 5 min 40 min	7	39 days/90 days/180 days: NIHSS, MoCA, ADAS‐Cog	Positive	Complete
Feng et al. (2019)^92^	14 days after disease onset	RIPostC	52/52	Healthy upper limb	200	–	1	5 × 5 min × 5 min 50 min	180	6 months: MoCA, ADL, HAMD, P300 latency (ms) and amplitude (μV), ICAM‐1, ET‐1	Positive	Complete
Zhou et al. 2019)^91^	Octo‐and Nonagenarians in sICAS	RIPostC	30/28	Bilateral upper arm	200	30	2	5 × 5 min × 5 min 50 min	180	300 days: WMHs volume, MMSE, MoCA, the Fazekas scale, Scheltens' scale, clinical symptoms	Positive	Complete
Wang et al. (2017)^90^	Patients with CSVD‐related mild cognitive impairment	RIC	14/16	Bilateral upper arm	200	50	2	5 × 5 min × 5 min 50 min	365	365 days: WMHs volume, MMSE, plasma biomarkers, cerebral hemodynamic parameters	Positive	Complete
Mi et al. (2016)^89^	Patients with CSVD	RIPreC	9/7	Bilateral upper arm	200	50	2	5 × 5 min × 5 min 50 min	365	Number of LIs, WMLs volume, MMSE, MoCA, DHI, cerebral hemodynamic changes	Negative	Complete
Patients with hemorrhagic stroke (HS)												
Zhao et al. (2022)^77^	Patients with supratentorial intracerebral hemorrhage presenting (STICH) within 24–48 h	RIC	20/20	Non‐affected arm	200	0	1	4 × 5 min × 5 min 40 min	7	3 days/7 days: hematoma volume, hematoma resolution rate, PHE volume 30 days/90 days/300 days: NIHSS, GCS, mRS, adverse event	Positive	Complete
Sangeetha et al. (2022)^76^	Patients with spontaneous subarachnoid hemorrhage (sSAH) scheduled for surgical clipping of the ruptured aneurysm	RIPreC	13/12	Bilateral upper arm	>SBP 30	30	Every 48 h	3 × 5 min × 5 min 30 min	7–10	Daily HR, BP, rScO2, GOSE, cerebral vasospasm	Positive	Complete
Jarrahi et al. (2022)^82^	Patients with sICH	RIC	10/10	Bilateral upper arm	200	0	2	4 × 5 min × 5 min 40 min	7	7 days/14 days: hematoma volume, functional outcomes, safety 30 days/90 days: mRS	No data	Ongoing
Sangeetha et al. (2021)^99^	Patients with aSAH scheduled for surgical clipping of the ruptured aneurysm	RIPreC	13/12	Bilateral upper arm	>SBP 30	30	Every 48 h	3 × 5 min × 5 min 30 min	7–10	S100B, NSE, GCS 30 days/90 days/180 days: GOSE	Positive	Complete
Raval et al. (2021)^75^	Patients with aSAH	RIPreC	11/11/11	Upper thigh	Not mentioned	>SBP 20 or <SBP 20	Every 48 h	4 × 5 min × 5 min 40 min	14	Cerebral vasospasm 30 days/90 days/180 days: mRS, hospital length of stay, mortality	Negative	Complete
Xu et al. (2020)^74^	Patients with aSAH	RIC	30/30	Two upper or lower limbs	180/>SBP 20	0	1	5 × 5 min × 5 min 50 min	7	PTA, PT, APTT, fibrinogen, D‐dimer, thromboelastogram, cerebral blood flow	Negative	Complete
Gonzalez et al. (2014)^73^	Patients with aSAH	RIC	20	Lower limbs	>SBP 20	–	NO	4 × 5 min × 5 min 40 min	1–13	Adverse events, GCS, mRS	Positive	Complete
Koch et al. (2011)^72^	Patients with aSAH	RIPreC	17/17	Two upper or lower limbs	200	30	Every 24–48 h	3 × 5 min × 5 min 30 min	14	VAS, GCS, mRS, tissue or neurovascular injury, symptomatic vasospasm	Positive	Complete

Abbreviations: ADAS‐Cog, Alzheimer's disease assessment scale‐cognitive section; ADL, activities of daily living; APTT, activated partial prothrombin time; bFGF, basic fibroblast growth factor; BI, measure of independence in activities of daily living; DHI, dizziness handicap inventory; EQ‐5D, Quality of Life Assessment Questionnaire; ET‐1, endothelin‐1; GCS, Glasgow Coma Scale; GOSE, Glasgow Outcome Score Extended; HAMD, Hamilton Depression Scale; HRV, heart rate variability; hs‐CRP, high‐sensitivity c‐reactive protein; HT, hemorrhagic transformation; ICAM‐1, intercellular adhesion molecule‐1; Lis, lacunar in‐fractions; MACE, major adverse cardiovascular events; MFV, mean flow velocity; MMP9, matrix metalloprotein‐9; MoCA, minimum obstruction clearance altitude; mRS, Modified Rankin Scale; NIHSS, National Institutes of Health Stroke Scale; OH1, heme oxygenase‐1; PGIC, patient global impression of change; PHE, perihematomal edema; PSV, peak systolic velocity; PT, prothrombin time; PTA, prothrombin activity; rCBF, regional cerebral blood flow; rMTT, relative mean transit time; SAP, serum amyloid protein; ScO2, cerebral oxygen saturation; sICH, symptomatic hemorrhagic transformation; TOI, tissue oxygenation index; VAS, Visual Analog Pain Scale; VEGF, vascular endothelial growth factor; WMLs, white matter lesions.

### 4.1 RIC for acute ischemic stroke

IVT or EVT therapy are the safest and most effective treatments for stroke as according to current guidelines.^4^, ^5^ Because the time window for effective treatment cannot exceed a maximum of 24 h, many people fail to receive treatment. Therefore, a safe and effective method that can be combined with conservative drug treatments to improve neurological impairment is RIC. Several studies have discovered that RIC can be applied to different time windows of AIS onset. The first time window occurs within 2–3 h immediately after RIC and may be associated with the production of endogenous substances by the therapeutic process, and the second phase occurs 12–24 h after RIC and can last 48–96 h or longer. The protective mechanism may be related to the release of endogenous substances mediating intracellular gene regulation and protein synthesis.^24^ RIC can protect the brain in the acute phase of ischemic stroke alone or in combination with EVT, which can also play a secondary prevention role in the recovery phase to avoid recurrence of cerebrovascular disease. RIC can also be applied to patients at high risk of stroke, such as those with intracranial stenosis or those who are suitable for endovascular recanalization therapy, with additional benefits such as reducing the incidence of stroke and disability.

#### 4.1.1 The safety and feasibility of RIC in hyperacute ischemic stroke patients within 6 h of onset

A study discovered that RIC was feasible in patients with AIS within 6 h of onset and did not increase the size of cerebral infarction.^25^ They enrolled 60 patients with AIS for RIC treatment and randomized them to a sham RIC group and a RIC treatment group, four cycles per day, given 1 day, 2 days, and 4 days of the treatment plan (RIC treatment, the unilateral upper arm was pressurized with a cuff for 5 min [20 mmHg above systolic blood pressure] and relaxed for 5 min, while the control arm underwent sham surgery with cuff expansion to only 30 mmHg). The results show that RIC is safe and neurologically injury‐free for hyper AIS within 6 h of onset and can be administered in repeated doses reliably for 2 days. Another French study was conducted in 188 patients with AIS within 6 h, 93 patients were randomly assigned to RIC (press on the thighs for 5 min to 110 mmHg above systolic pressure, then relax for 5 min for four sets) of the lower extremity in addition to standard care, which showed RIC had no significant effect on the increase in cerebral ischemic volume 24 h after symptom onset.^26^

#### 4.1.2 The safety and feasibility of RIC in AIS patients within 24 h of onset

England et al.^27^ found that RIC is safe and feasible for AIS patients with onset time less than 24 h. This study included 26 AIS patients who had not received IVT or endovascular treatment within 24 h. A blood pressure cuff was used on the nonparalyzed arm with four periods of inflation for 5 min and deflated for 5 min (20 mmHg above SBP) and 5 min of release. The control group received sham treatment (pressure of 30 mmHg). The results showed that RIC improved neurological prognosis, reduced the incidence of vascular events, significantly improved NIHSS scores, and reduced vascular events at Day 90. The protective mechanism may be mediated by heart shock protein 27 (HSP27). Serum HSP27 was markedly higher in the RIC group than in the control group. and HSP27 has a neuroprotective effect and reduces the volume of cerebral infarction. In 2021, based on the same trial,^28^ the team found that RIC reduced SAP (serum amyloid protein) and TNF (tissue necrosis factor) levels, and SAP levels were directly proportional to the prognosis of brain injury in AIS patients. Landman et al.^29^ found that no significant improvement in the final infarct size or clinical outcome when patients with AIS with onset within 24 h were given RIC. The test method was that adult patients with AIS were randomly distributed to repeat RIC or sham conditioning at a 1:1 rate. RIC was repeated by inflating a blood pressure cuff (4 × 5 min at 200 mmHg) around the upper arm twice a day for up to 4 days during the hospital stay. This is a possible correlated to these conclusions as the study's patient inclusion rate was too low, there were also baseline differences in NIHSS scores between the two groups, and the mean cerebral infarct size of the patients was relatively small, with no infarct lesions visible on MRI in 19% of the patients. This ultimately led to potentially biased results, and large‐scale trials are still needed to validate the effectiveness of RIC in the future.

#### 4.1.3 The efficacy of RIC treatment in AIS patients within 48 h of onset: evidence from multiple studies

Multiple researchers have proved that RIC treatment is feasible and safe when the onset time window of AIS patients is extended to 48 h or even beyond which can effectively improve the neurological deficits of AIS patients and have a good prognosis. A multicenter RCT (NCT03740971) with a large‐sample size from China provides important evidence for the efficacy of RIC in AIS patients.^30^ They included a total of 1893 patients who experienced acute and moderate AIS within 48 h and NIHSS scores between 6 and 16, excluding patients who received IVT and EVT. The cuff of the RIC medical device was placed around the bilateral upper limbs, five cycles of alternating ischemia for 5 min (200 mmHg) and reperfusion for 5 min, the duration is 50 min, twice a day for 10–14 days. The control group was only given standard guideline‐based therapy. The results indicated that the RIC group had positive results at 90 days (risk difference, 5.4% [95% CI, 1.0%–9.9%]; odds ratio, 1.27 [95% CI, 1.05–1.54]; *p* = 0.02). This suggests the conclusion that treatment with RIC compared with guideline‐recommended treatment alone increased the likelihood of recovery of neurological function after 90 days. Another study also included AIS patients who had not received IVT or EVT within 48 h of onset.^31^ Patients were randomly classified into RIC group and control group. Based on the same drug treatment, RIC treatment (180 mmHg) and sham RIC treatment (60 mmHg) were given respectively. The arm was pressurized for 5 min using a blood pressure cuff and then relaxed for 3 min, with each cycle consisting of five I/R cycles lasting 40 min. twice daily for 7 days. The results show that compared with the control group and conventional drug therapy, RIC is effective and safe in the treatment of AIS and can alleviate neurological deficits in AIS patients. RIC does not affect the platelet function of patients and can be used as adjuvant therapy for combined antiplatelet therapy in AIS patients.

#### 4.1.4 The efficacy of RIC treatment in AIS patients within 72 h of onset

A study demonstrated the safety and efficacy of RIC in patients with AIS within 72 h of onset and suggested that RIC may improve neurological recovery through a protective mechanism of increasing cerebral blood flow to improve cerebral perfusion.^32^ All enrolled patients were randomly divided into RIC group (20 mmHg above systolic BP) and a control group (cuff inflation to 30 mmHg) and treated with I/R (5 min of pressure and 5 min of relaxation with a blood pressure cuff on the nonparalyzed arm) once a day for 14 consecutive days. After 90 days, compared with controls, RIC significantly decreased NIHSS scores, regional relative mean transit time (*p* < 0.05), increased cure rate of mRS, regional relative cerebral blood flow (*p* < 0.05), and reduced cerebral ischemic volume by 31.3% (*p* < 0.05). Another study found that RIC improved autonomic function by enhancing both autonomic and vagal activity in AIS patients after 30 days of continuous use within 14 days of onset.^33^

#### 4.1.5 RIC in acute ischemic stroke treatment: current status and future directions

Based on the above studies, most trials have proved that RIC therapy is safe and effective whether it is applied before, during, or after the arrival of AIS patients. RIC can play a protective role in AIS at different time windows. This is especially important given the narrow window of traditional post‐AIS intervention. Although two studies have shown that RIC did not decrease infarct size in AIS patients with onset within 6 h and 24 h, respectively, it may be because the RIC treatment cycle is too short, or the follow‐up time is too close to the onset time. Therefore, a study of RIC applied to adults with suspected AIS symptoms within 8 h is ongoing (NCT03375762).^34^ The treatment plan of this study is as follows: RIC will be initiated in an ambulance before hospitalization and sustained in the hospital, RIC is applied by placing the tourniquet to the arm of the unaffected side for five cycles, each consisting of 5 min of pressurization (200 mmHg pressure) and 5 min of relaxation. No pressure was applied in the control group. The objective is to observe the function of RIC on improving the neurological function of AIS patients. Furthermore, a study of early RIC combined with exercise (RICE) therapy within 24 h is underway in China.^35^ They will enroll AIS patients within 24 h of symptom onset or deterioration and randomly divide them into the RIC group (five cycles of pressurization of 200 mmHg for 5 min followed by relaxation for 5 min) and control group (same method of pressurization of 60 mmHg), 1 time daily for 14 days. All participants will receive under‐bed exercise training for 30 min twice daily for 11 days starting 4 days after the onset of symptoms. The main endpoint events are safety and incidence of adverse events, and the secondary result is prognosis. A Japanese study is currently underway on the effectiveness of RIC in treating AIS patients within 48 h of symptom onset.^36^ The primary outcome will be evaluated by the mRS score 90 days after stroke onset. In the future, more large‐scale multicenter trials are needed to prove the optimal time window and the most effective application regimen of RIC treatment in patients with AIS, and it is necessary to confirm the brain‐protective effect of RIC treatment in patients with AIS by different metrics such as imaging or blood markers. At the same time, it is better to extend the follow‐up time after RIC treatment and reduce the proportion of patients who lose follow‐up to provide more reliable evidence.

#### 4.1.6 RIC combined with intravenous thrombolysis or endovascular thrombectomy

Currently, the use of recanalization therapy (IVT and EVT) in the acute phase of AIS is the most effective and safe treatment for AIS patients, and it can achieve better clinical functional outcomes compared with conventional medical therapy.^4^, ^5^ However, IVT treatment may disrupt the blood–brain barrier (BBB), resulting in brain edema and ICH,^37^, ^38^, ^39^ and only about 31% of IVT patients achieved a good stroke outcome (mRS score of 0 or 1) at 3–6 months.^5^ Although approximately 80% of vascular occlusions can be recanalized by EVT, only 46% of patients treated with EVT have a good prognosis after 90 days (mRS Score, 0–2), and more than 50% of patients die after 90 days of treatment.^40^, ^41^ Poor prognosis may be due to incomplete recanalization or early arterial reclusions after recanalization therapy.^42^ Therefore, several researches have investigated the safety and efficacy of RIC combined with recanalization therapy. In 2013, Hougaard et al.^13^ were the first to randomize RIC to patients with suspected AIS in the ambulance before admission, patients who subsequently underwent IVT were selected. The RIC group used the cuff to perform four cycles of alternating pressure for 5 min (pressure of 200 mmHg or >BP 25 mmHg) and relax for 5 min. The results suggest that pre‐hospital RIC treatment as an adjunct to intravenous rt‐PA is safe, but the overall results were neutral. RIC treatment had no significant effect on infarct growth, final infarct size, penumbral salvage, and clinical outcome at 90 days. Further analysis revealed that RIC reduced tissue risk of infarction (*p* = 0.0003), and this suggests that RIC may have direct neuroprotective effects. Patients who received RIC had notably lower NIHSS scores and reduced cerebral hypoperfusion and brain tissue damage compared with non‐RIC group. Subsequently, scientists began to review the safety and availability of RIC after IVT treatment in AIS patients. In 2018, a Chinese study^43^ enrolled 30 AIS patients undergoing IVT and randomly classified them into two groups on a 1:1 basis. Participants in both groups received standard medical care. In addition to the above treatment within 2 h after IVT, the patient's bilateral/single upper limbs were alternately inflated five times with a pressure of 200 mmHg and then inflated for 5 min, twice daily for 6 days starting the next day. The results showed that the noticeable decrease in NIHSS scores on Day 30 in the RIC group (*p* = 0.037). This demonstrates the safety and availability of RIC combined with IVT therapy in patients with AIS, and it is well tolerated within an appropriate time window. In the same year, the team members found that RIC combined with EVT was also safe and feasible for AIS patients.^44^ They applied RIC to patients with AIS who had an anterior circulation large vessel occlusion within 6 h of onset, and performed four cycles of RIC before recanalization, immediately after recanalization, and once daily for the next 7 days. The findings indicated that RIC was well‐safe and tolerated, with no significant negative effects on intracranial pressure, cerebral perfusion pressure, mean arterial pressure, or heart rate. In 2020, a study enrolled 49 AIS patients who were treated with IVT and received two RIC treatments within 6–24 h of IVT, each group received 5 min of inflation and 5 min of deflation in the unilateral upper limb for four cycles (RIC group: 200 mmHg, sham RIC: 60 mmHg). The findings indicated the safety of RIC in conjunction with IVT treatment. C‐reactive protein (hs‐CRP) levels were meaningfully lower in the RIC group compared with the control group (*p* = 0.048), suggesting that RIC may have an anti‐inflammatory effect.^45^ Another study included 68 AIS patients receiving IVT who were randomized to RIC or control groups.^46^ During the hospitalization, RIC was administered twice daily to both upper arms, each consisting of five cycles of compression (to 180 mmHg for 5 min) and relaxation (for 3 min), the control group underwent no inflations or deflations. RIC was administered for 8–14 days, with an average duration of 11.2 days. The outcomes demonstrated that the symptom improvement rate of patients in the RIC treatment group was meaningfully higher than the control group (71.9% vs. 50%, *p* = 0.016). It was also found that RIC may promote the recovery of neurological function in AIS patients through plasma S100 β and vascular endothelial growth factor. Previous research has shown the safety and availability of RIC in AIS patients receiving IVT or EVT. However, the effectiveness of RIC in combination with IVT or EVT needs to be further determined. To address this, two large multicenter clinical trials are currently underway to study RIC in combination with IVT (NCT04980625) or EVT (NCT04977869),^47^ which could provide clinical evidence for this approach. However, there are several challenges with previous studies that need to be addressed. Firstly, the narrow treatment window for IVT or EVT means that RIC treatment before recanalization may not be sufficient to achieve the desired therapeutic effects. Therefore, it is best to start RIC treatment on the emergency vehicle before admission if possible. Secondly, large‐scale clinical trials are needed to further investigate the cycle, frequency, and pressure of RIC combined with IVT or EVT treatment if RIC is used after vascular opening. Finally, the small sample size of previous trials highlights the need for more large‐scale multicenter trials to provide the most reliable and authentic evidence for RIC combined with IVT or EVT treatment in the future.

### 4.2 The effectiveness of RIC in treating patients with symptomatic intracranial atherosclerotic stenosis

It is well known that ischemic stroke is characterized by a high recurrence rate, especially intracranial atherosclerotic stenosis type (ICAS).^48^ The risk of recurrence in patients with ICAS within 2 years after stroke can be as high as 25%–30%.^48^, ^49^ At present, the main methods to prevent the recurrence of ischemic stroke are antiplatelet aggregation therapy and control of risk factors.^50^ While EVT such as interventional stenting has been regarded as the most promising treatment for ICAS patients, recent studies have shown that in patients with severe ICAS, there is no significant difference in the risk of stroke or death after 30 days to 1 year of treatment with drugs combined with percutaneous transluminal angioplasty and stenting compared with drug therapy alone.^51^ Surgical treatment also carries a risk of perioperative complications. Therefore, some scholars began to explore neoadjuvant therapy to prevent the recurrence of stroke.

Previous researches have demonstrated the neuroprotective effects of RIC in patients with AIS. To demonstrate the safety of RIC in patients with symptomatic intracranial atherosclerotic stenosis (sICAS), a research team compared RIC in patients with unilateral middle cerebral artery (MCA) stenosis with healthy volunteers,^52^ all of whom received five cycles of RIC (200 mmHg pressurized for 5 min and then deflated for 5 min). The results showed no major effect of RIC on heart rate, oxygenation index, and mean blood flow in both groups of subjects and that it is safe and tolerable. To further investigate the effectiveness of RIC in treating sICAS patients, the team conducted two clinical trials, in which sICAS patients under 80 years old were treated with RIC for 300 days (NCT01321749)^15^ and sICAS patients over 80 years old were treated with RIC for 180 days (NCT01570231),^14^ twice a day in both cases (inflate the cuff to 200 mmHg for 5 min and then deflate for 5 min for a total of five cycles). The results show that RIC therapy is not only effective for sICAS patients under 80 years of age, but also for octo‐and nonagenarians. It can prevent the occurrence and recurrence of stroke, and promote neurological recovery in elderly sICAS patients, possibly by improving cerebral perfusion and plasma biomarkers that reduce inflammation and clotting in sICAS patients.^14^, ^15^ Based on these trials, another study evaluated the effectiveness of RIC treatment on the rehabilitation of nerve function and collateral circulation in young patients (age: 18–45, gender‐balanced) with sICAS.^53^ They recruited 100 young sICAS patients randomly grouped, with the control group receiving only medication and the RIC group receiving RIC twice a day for 6 months on top of medication. At present, the study has confirmed the safety of repeated RIC treatment in patients with sICAS, but the role of collateral circulation rehabilitation is still being researched. In 2021, a high‐volume single‐center prospective cohort study from China was published to investigate the effect of chronic RIC treatment on patients with symptomatic internal carotid artery (ICA) or middle cerebral artery (MCA) occlusion.^54^ In the 10‐year cohort study, 664 patients with ICA or MCA occlusion were enrolled. All patients were treated with RIC once or twice daily (cuff pressure of 200 mmHg, 5 min of pressure followed by 5 min of relaxation for five cycles). Excluding those patients without hemodynamic compromise or asymptomatic, non‐atherosclerotic disease, and those with moyamoya disease or surgically treated disease, a total of 131 patients were ultimately recruited for inclusion in the study, with a mean follow‐up of 8.8 years. The results showed a compliance rate of 95.6 ± 3.7% for RIC, an annual risk of AIS and ischemic cerebrovascular events of 2.4% and 3.3%, respectively, and no RIC‐related adverse events. After 14 years, the probability of an ischemic cerebrovascular event was 32.8% and the cumulative probability of a major adverse cardiovascular event was 44.8%. In conclusion, chronic RIC therapy is well tolerated in patients with ICA or MCA occlusion and can reduce the incidence of cardiovascular and cerebrovascular events. In addition, a single‐arm study (PICNIC‐One Study)^55^ found that continued application of RIC for 90 days reduced the risk of high‐risk, non‐disabling ischemic cerebrovascular events. A large multicenter RCT trial in China (RICA, clinicaltrials.gov NCT 02534545), provides high‐level medical evidence for RIC treatment of ICAS.^56^ A total of 3033 participants (ischemic stroke within 30 days or TIA within 15 days) were randomly distributed to RIC or control groups, all participants received their usual drug therapy. The RIC group will be inflated for 5 min (pressure of 200 mmHg) and deflated for 5 min for five cycles. The sham RIC is treated with an inflation pressure of 60 mmHg. The median follow‐up period was 3.5 years. The outcome indicates that the RIC group significantly reduced the recurrence rate of stroke compared with the control group (14.7% vs. 18.7%, *p* = 0.038), and the long‐term RIC treatment significantly reduced the incidence of cardiovascular and cerebrovascular events (17.6% vs. 24.1%, *p* = 0.0026). Therefore, chronic RIC treatment can effectively prevent the recurrence of stroke and reduce the occurrence of cardiovascular and cerebrovascular diseases.

### 4.3 A potential adjuvant therapy for carotid artery stenting and carotid endarterectomy

RIC therapy is not only safe and effective for ICAS patients but may also be used as an adjuvant therapy for carotid artery stenting (CAS) and carotid endarterectomy (CEA) in ICAS patients. In 2017, Zhao et al.^57^ enrolled 189 patients with severe ICAS who were preparing for CAS and were randomly divided into three groups at a 1:1:1 rate (RIC group: The cuff was tied to both upper arms at a pressure of 200 mmHg, followed by five cycles of alternating 5 min of compression and 5 min of relaxation twice daily for 14 days; sham group: same as above, but with cuff pressure of 60 mmHg; control group: standard medication treatment). The results showed that the incidence of new lesions was obviously lower in the RIC group (15.87%) than in the prosthesis group (36.51%, *p* < 0.01) and the control group (41.27%, *p* < 0.01). The lesion volume of the RIC group was obviously lower than that of the sham group and the control group (*p* < 0.01 each). The conclusion that can be drawn is that daily use of RIC for 2 weeks before CAS treatment is safe and feasible; it can effectively reduce secondary brain injury, and has good tolerability and high compliance. Two other trials evaluated the effect of RIC therapy in patients with CEA. To evaluate whether RIC affects nerve or heart injury after CEA, Walsh et al.^58^ included 70 patients who received CEA treatment and were randomly classified into the RIC group or control group. Patients in the RIC group were given RIC treatment for 10 min once in both lower limbs after operation anesthesia and before recanalization, while patients in control group were only given conventional treatment. RIC combined with CEA seems to be safe with no adverse events, but the clinical benefit needs to be verified in large‐scale trials. Another trial examined the efficacy and safety of RIC in macrovascular surgery.^57^ They included 198 patients undergoing macrovascular surgery who were randomly assigned 1:1 to the RIC combination or control group, which received four cycles of 5 min of pressure by 5 min of relaxation at a pressure of 200 mmHg. Among them, 45 patients underwent CEA (RIC group: 24 people; Control: 21 people), the results showed that RIC combined with CEA had no significant effect, and the difference was not statistically significant (*p* = 1.00). The current study confirms that RIC therapy in combination with CEA is well tolerated and safe. However, the efficacy of RIC, the optimal treatment regimen, and the mechanism of neuroprotective effects still need to be further investigated in large‐scale randomized controlled trials.

### 4.4 The potential role of RIC in the treatment of moyamoya disease

Moyamoya disease (MMD) is a progressive, irreversible stenosis, or occlusive disease of the cerebral vasculature that predisposes the patient to thrombosis and hemorrhage because of abnormal cerebral vascular development.^59^, ^60^ The current treatment is mainly revascularization surgery.^61^ However, the perioperative period may cause postoperative complications such as hyperperfusion syndrome.^62^ RIC can be used as an adjunctive treatment for patients with MMD, reducing the recurrence rate of ischemic events and improving cerebral blood volume. In 2019, Ding et al.^63^ performed a long‐term RIC study in 30 patients with MMD (five cycles of alternating compressions for 5 min and relaxation for 5 min). The outcomes indicated that the recurrence rate of stroke was obviously reduced after RIC (*p* = 0.013), which relieved symptoms and improved cerebral perfusion. In 2022, Xu et al.^64^ included 34 patients with MMD, randomly classified into RIC and control groups, both groups were given conventional drug therapy, and the RIC group was treated with RIC continuously for 1 year on top of the above treatment. The outcomes demonstrated that the mean cerebral blood flow (mCBF) improved more meaningfully in the RIC group than in the control group (*p* = 0.001), and RIC improved cerebral blood volume and slowed the progression of cerebrovascular stenosis. Another study^65^ investigating the safety and efficacy of RIC in pediatric MMD patients undergoing revascularization surgery is underway. The primary outcome was evaluation of the cerebral perfusion status at 3 months postoperatively as assessed by single‐photon emission computed tomography (SPECT). Secondary outcomes were safety of RIC, brain injury biomarkers (S‐100A4, MMP‐9), and vascular biomarkers, as well as functional outcomes, incidence of cerebral infarcts, and death and adverse event.

Based on the above research, RIC therapy can effectively reduce ischemic stroke incidence, especially in the recurrence rate of ICAS patients, improve cerebral blood flow perfusion, and enhance cerebral nerve recovery. Chronic RIC can reduce the incidence of cardiovascular and cerebrovascular diseases. RIC also improves cerebrovascular volume and reduces the incidence of ischemic events in patients with MMD. It is expected that more large‐scale trials will be conducted in the future to specifically explore the ways in which RIC therapy could be used to prevent ischemic stroke recurrence, and to better incorporate blood markers or imaging findings into clinical outcomes. At the same time, the current trials have shown that RIC combined with CEA is safe and well tolerated, but more large‐scale randomized controlled trials are necessary to prove the effectiveness of RIC in combination with CEA, explore the best treatment plan of RIC application and preoperative or postoperative time, cycle and pressure, and improve the compliance and follow‐up rate of subjects, to provide strong evidence for RIC treatment in prevention of the recurrence of ischemic stroke.

### 4.5 RIC treatment of hemorrhagic stroke

Hemorrhagic stroke comprises around 20% of all strokes including aSAH and ICH. HS is associated with severe morbidity and high mortality.^66^ Progression of HS is associated with worse outcomes as compared to ischemic stroke. Around 40%–70% of aSAH patients develop cerebral vasospasm and delayed cerebral ischemia (DCI) 4–10 days after surgery, which are the main causes of poor neurological prognosis. Intracerebral hemorrhage has a mortality rate of up to 50% at 30 days, and its adverse outcome is mainly hematoma subsequent perihematomal edema (PHE). Hematoma removal and remission of PHE are the key to the treatment of ICH.^67^, ^68^ At present, there is no effective treatment that can significantly improve the prognosis of HS patients. Many interventions for the treatment of HS have been investigated in recent years, such as invasive surgery to remove hematomas.^69^ However, some studies have demonstrated that the clinical benefit of surgery after cerebral hemorrhage is unclear.^70^ Minimally invasive surgical removal of hematoma may reduce hematoma volume, but the clinical function has no obvious improvement.^71^ Therefore, it is extremely essential to explore safe, noninvasive, effective, and controllable neuroprotection technology. Some researchers have found that RIC therapy may be safe and effective as a noninvasive neuroprotective strategy for patients with HS, and RIC may also provide adjunctive therapy to minimally invasive surgical hematoma removal. Koch et al.^72^ enrolled 33 awake aSAH patients and performed RIC treatment on the upper limb every 24–48 h, and there were no statistically significant differences between the groups for any of the baseline characteristics. The outcomes indicated that RIC was safe, feasible, and well tolerated in the treatment of aSAH. Gonzalez et al.^73^ did a small sample non‐RCT study in which aSAH patients who met the inclusion criteria were given several courses of RIC. The patient's leg was given 4 periods of pressure for 5 min and relaxation for 5 min at a pressure of 200 mmHg. The outcomes indicate that RIC was well tolerated in aSAH patients without any injury. To observe the influence of RIC on coagulation function and cerebral blood flow in aSAH patients, Xu et al.^74^ recruited 30 patients with aSAH to compare the changes before and after the patients applied RIC therapy. They were required to receive RIC for 7 consecutive days during their hospitalization (compression of 180 mmHg or >BP20 mgHg in bilateral upper or lower limbs, followed by 5 min of ischemia and 5 min of reperfusion in five cycles). The findings indicated that RIC had no notable impacts on coagulation function and cerebral blood flow in aSAH patients, and no deep vein thrombosis and cerebral infarction were found. Raval et al.^75^ included 22 patients with aSAH within 72 h of onset and randomized into high‐pressure occlusion group (>SBP 20) and low‐pressure occlusion group (<SBP 20), with an additional 11 patients from retrospectively matched cohort as controls. Patients were treated with four periods of 5 min of compression followed by 5 min of relaxation for a total of 14 days. The outcomes revealed that there were no statistically significant outcomes such as GCS score, days of vasospasm, mRS Score, and adverse events. The duration of hospitalization and ICU stay was shorter in the low‐pressure occlusion group compared with the control group. It is demonstrated that RIC is safe and viable for treating patients with aSAH, but the effectiveness of RIC still needs to be further verified. Sangeetha et al.^76^ found that RIC may affect the regional cerebral oxygen saturation (rScO2) of aSAH patients. They recruited 25 aSAH patients who were scheduled to undergo aneurysm clipping within 72 h and were randomly classified into the RIC group (*n* = 13) and the sham‐RIC group (*n* = 12), baseline demographic parameters were comparable between the groups. RIC was administered every 48 h (three cycles of ischemia, 5 min of relaxation, 5 min of relaxation, pressure > BP 30 mmHg) or sham‐RIC treatment (same procedure, pressure = 30 mmHg) for about 7–10 days. The findings indicate that the incidence of cerebral oxygen desaturation in RIC group was decreased compared with baseline level (15.4% vs. 33.3%, *p* < 0.05; *p* = 0.378). The RIC group had a higher mean Glasgow outcome scale extended compared with sham group (estimate 0.8, *p* = 0.027). From this, it is clear that RIC may play a cerebral protective role by affecting cerebral oxygen desaturation in patients with aSAH.

Based on the above study, Zhao et al.^77^ found that RIC may also be safe and well tolerated in ICH patients. A total of 40 patients with supratentorial ICH presenting within 24–48 h were randomly classified into RIC group and control group. The control group received standard treatment according to the guidelines, while the RIC group based on standard treatment received four cycles of pressure on the unilateral upper limb for 5 min followed by 5 min of relaxation, pressure = 200 mmHg, twice a day for 7 days. The outcomes indicate that none of the patients had neurological deterioration or adverse events related to RIC within 7 days. After 7 days, there were no statistically considerable changes in hematoma volumes between the RIC and control groups compared with baseline (*p* > 0.05 each), but the rate of hematoma resolution was obviously higher in the RIC group compared with the control group (49.25 vs. 41.92, *p* = 0.015). The relative perihematomal edema in the RIC group was meaningfully lower than in the control group (1.77 vs. 2.02, *p* = 0.023). It can be concluded that RIC therapy is safe and tolerable for ICH patients, and it may improve the rate of hematoma dissolution and reduce hematomas. It is well known that hematoma volume in patients with cerebral hemorrhage is independently associated with severity and prognosis.^78^, ^79^ Hematoma clearance is considered the most critical treatment for ICH and cerebral hemorrhage. This study demonstrated that repeated RIC for 7 consecutive days in patients with ICH significantly increased the rate of hematoma resolution as patients in the RIC group showed a reduction in hematoma volume of more than 14.4% compared with the control group.^80^ The higher hematoma resolution rate may also indicate the promoting effect of RIC on hematoma resolution in patients with ICH. In addition, a clinical trial (RESIST, NCT03481777)^81^ and another randomized controlled trial^82^ are currently underway to further investigate the safety, efficacy, and practicability of RIC in ICH patients.

At present, RIC is mainly applied to ischemic stroke, and there are not many trials on HS, and most of them focus on aSAH diseases, and few reports on ICH diseases. The above trials have proved the safety, practicability, and tolerability of RIC in HS, but the effectiveness of RIC in the treatment of HS needs to be further verified. In addition, the above tests also have certain limitations, such as insufficient sample size, patients lost to follow‐up, and unclear brain‐protective effect. As aSAH and ICH share many pathophysiological mechanisms, larger preclinical and clinical trials are needed in the future to delineate the neuroprotective mechanism, effective approach, and optimal treatment regimen of RIC for HS patients, to provide more authoritative and credible evidence for RIC treatment.

### 4.6 RIC improves vascular cognitive impairment

#### 4.6.1 Effect of RIC on vascular cognitive impairment

Vascular cognitive impairment is a leading cause of cognitive dysfunction and dementia in elderly individuals. Two common forms of VCI are cerebral small vessel disease (CSVD) and poststroke cognitive impairment (PSCI).^83^ Cerebral small vessel disease mainly affects the white matter and deep structure of the brain. Imaging findings mainly include white matter hyperintensity (WMHs), subcortical cerebral infarction, cerebral atrophy, or cerebral microhemorrhage.^84^ Cerebral small vessel disease is reported to be responsible for 25% of strokes and 45% of cognitive impairment events.^85^ The prevalence increases with age, affecting approximately 50% of people over 50 years of age and nearly 100% of people over 90 years of age.^86^ Poststroke cognitive impairment is a common complication after AIS and affects approximately 41% of patients 1 year after stroke.^87^, ^88^ Recent studies have also shown that RIC can improve cognitive function, reduce the degree of cerebral arteriosclerosis, and improve cerebral blood flow in stroke patients with PSCI.

#### 4.6.2 Effect of remote ischemic conditioning on vascular cognitive impairment

Mi et al.^89^ did a pilot RCT involving 17 patients with CSVD who were randomly classified into RIC group and control group. Five periods of 5 min of ischemia and 5 min of reperfusion were administered in bilateral upper arms twice a day for 12 months at a pressure of 200 mmHg in the RIC group and 50 mmHg in the control group. The findings demonstrated that there was no obviously difference in WMHs between RIC group and control group, but the volume of WMHs in RIC group was significantly reduced before and after treatment (4.19 mL vs. 6.06 mL, *p* = 0.050), the mean MCA flow rate of patients after RIC treatment was significantly increased compared with that before treatment (57.33 cm vs. 51.33 cm, *p* = 0.038). However, there was no obvious difference in cognitive function (MMSE and MoCA scores). Based on the above studies, Wang et al.^90^ further evaluated the therapeutic use of RIC in treating patients with CSVD combined with mild cognitive impairment. They recruited 30 subjects and randomly assigned them to the RIC group (*n* = 14) or the control group (*n* = 16) for 12 months (the method is the same as the above experiment). The findings indicated that the volume of WMHs in RIC group was obviously reduced compared with that in the control group (2.632 mL vs. 0.935 mL, *p* = 0.049). After 1 year of treatment, there was a significant difference between the two groups in terms of visuospatial and executive abilities (0.639 vs. 0.191; *p* = 0.048). RIC may effectively alleviate the progression of cognitive impairment and white matter damage in patients with CSVD. The same team found that RIC may also improve cognitive impairment and prevent the progression of WMHs in very elderly ICAS patients.^91^ Fifty‐eight patients with ICAS were randomly classified into RIC group (*n* = 30) or control group (*n* = 28). RIC (200 mmHg) and sham RIC (30 mmHg) were administered to both upper arms with five cycles of alternating between 5 min of expansion and 5 min of deflation, twice daily for 300 days. The results showed that RIC treatment obviously reduced WMH changes at 180 days (*p* < 0.05) and 300 days (*p* < 0.01) of follow‐up compared with baseline data. Compared with the control group, the RIC group had meaningfully lower WMHs changes at 300 days (*p* < 0.001). Remote ischemic conditioning significantly improved the MMSE score and MoCA score at 180 and 300 days of follow‐up, with statistically meaningful differences between the two groups compared with the control group (all *p* < 0.001).

#### 4.6.3 The safety and efficacy of RIC in the treatment of PSCI

Other researchers have evaluated the safety and efficacy of RIC in the treatment of PSCI. Feng et al.^92^ enrolled 104 patients with non‐cardiogenic AIS who were randomly classified into two groups (control group: standard therapy; RIC group: standard therapy five cycles of compression for 5 min and relaxation for 5 min at a pressure of 200 mmHg for 6 months). The outcomes indicated that after 6 months, compared with the control group, the total score of MoCA and domains of visuospatial and executive functioning and attention scores of the RIC group were significantly improved (*p* < 0.05), and activity of daily living scale (ADL) was obviously decreased (*p* < 0.05). The patients in the RIC group had a shorter P300 latency (on average) and higher wave amplitude than the control group (*p* < 0.05). The average blood flow velocity of MCA in the RIC group was significantly higher than that in control group (*p* < 0.05). This suggests that RIC can reduce the degree of cerebral arteriosclerosis, improve cognitive function, and reduce the incidence of PSCI by improving cerebral blood flow and endothelial function in stroke patients. Li et al.^32^ recruited 48 patients with AIS and randomly assigned them to RIC or sham RIC for 7 days, each with RIC (200 mmHg) or sham RIC (30 mmHg). MoCA and Alzheimer's disease assessment scale‐cognitive (ADAS‐Cog) were used to evaluate cognitive function. The findings indicate that the MoCA score of RIC group at Day 90 and Day 180 was obviously higher than that of control group (*p* < 0.05). The ADAS‐Cog score improved significantly on Day 180 (*p* < 0.05). Therefore, RIC treatment is safe and feasible for patients with PSCI and may improve cognitive function. Poalelungi et al.^93^ found that 5 consecutive days of RIC treatment in AIS patients within 24 h of onset resulted in a slightly higher MoCA score after 6 months in the RIC group than in the control group, but the results were not statistically significant. Therefore, more and larger scale trials evaluating the efficacy of RIC for PSCI are needed in the future to draw definite conclusions.

The above evidence gives us reason to believe that RIC is safe and available in the treatment of vascular cognitive dysfunction, possibly by reducing the volume of WNHs, improving cerebral blood flow, and reducing the degree of arterial stiffness to play a brain‐protective role. Since VCI is a long‐term chronic disease with a complex pathogenesis, the above tests have limitations such as small sample size and limited follow‐up time. Therefore, future large‐scale multicenter randomized clinical trials are required to further discover the mechanism and effective approach of RIC in the treatment of VCI, as well as the efficacy of early and long‐term use of RIC. Considering VCI as a chronic disease, we should consider how to improve the duration and compliance of RIC use in subjects.

## 5 CONCLUSION

Remote ischemic conditioning has great potential as an innovative therapeutic approach in the treatment of stroke. It can protect the brain in the acute phase of ischemic stroke alone or in combination with EVT and can play a secondary prevention role in the recovery phase to avoid recurrence of cerebrovascular disease. It is also well tolerated in HSs. However, the clinical application and basic research of RIC are still in the preliminary stage of study, such as the optimal time and period of application of RIC I/R and specific mechanism of action still need to be further explored. It is expected that more high‐quality, large‐sample, multicenter RCTs will be reported around the world in the future. As the problems are solved one by one, RIC will bring a breakthrough in stroke prevention and treatment. With all the potential applications of RIC, it might 1 day be possible to use it as a preventative measure in high‐risk populations.

## AUTHOR CONTRIBUTIONS

Bin Jiang designed the study, collected and analyzed the data, and worked on the manuscript. Xiaojie Wang and Jianping Ma were involved in the design of the study. Yuchuan Ding and Aminah Fayyaz contributed mainly to discussions of its content, and reviewing and editing the manuscript prior to submission. Pei Qin and Li Wang participated in analyzed the data. Sijie Li and Xunming Ji are the corresponding author and participated in the design and coordination of this study. All authors contributed to the article and approved the submitted version.

## CONFLICT OF INTEREST STATEMENT

The authors declare that they have no competing interests.

## Data Availability

The data that support the findings of this study are available from the corresponding author upon reasonable request.
